# Purification of total flavonoids from *Rhizoma Smilacis Glabrae* through cyclodextrin‐assisted extraction and resin adsorption

**DOI:** 10.1002/fsn3.809

**Published:** 2019-01-29

**Authors:** Li Zhang, Dan Zheng, Qing‐Feng Zhang

**Affiliations:** ^1^ Jiangxi Key Laboratory of Natural Product and Functional Food College of Food Science and Engineering Jiangxi Agricultural University Nanchang China

**Keywords:** astilbin, cyclodextrin, macroporous resin, *Rhizoma Smilacis Glabrae*, total flavonoids

## Abstract

Flavonoids are the main bioactive components responded for the health promoting effects of *Rhizoma Smilacis Glabrae* (*RSG*), an herbal material used in many functional food of China. An eco‐friendly method with β‐cyclodextrin (β‐CD)‐assisted extraction and resin adsorption/desorption was developed for total flavonoids purification from *RSG*. Because of complexes formation between flavonoids and β‐CD, aqueous solution was used for extraction instead of ethanol. The CD‐assisted extraction was optimized through defining optimal CD species, concentration, extraction temperature, and time. The adsorption property of eight macroporous resins on astilbin was compared by adsorption kinetics and capacity. All resins could reach the adsorption equilibrium within 2 hr. Further analyzed by Langmuir and Freundlich models, H103 resin with the best adsorption capacity was selected. The desorption property of different ethanol–water solution was compared. Results showed that by using 75% ethanol, astilbin could be well desorbed from the resin with the recovery of 96.3%. Because of complexes formation, the presence of β‐CD would slightly decrease the adsorption rate and capacity of H103 with concentration dependent. In dynamic adsorption, decreasing the flow rate could minish the effects of β‐CD. The developed method was successfully used for total flavonoids purified from *RSG*. The yield of purified product was 8.78%, with astilbin and total flavonoids content of 363.8 and 505.7 mg/g, respectively. The purity was 1.74 times increased with the recovery of 94.38% compared with the extract obtained directly through 50% ethanol extraction.

## 1 INTRODUCTION

*Rhizoma Smilacis Glabrae* (*RSG*) is an herbal material commonly used in functional food and Traditional Chinese medicine. Modern pharmacological studies have showed that the extract of *RSG* has the bioactivities of antioxidative (Zhang, Li, Lai, & Cheung, 2009; Zhang, Zhang, & Cheung, 2009), anti‐inflammatory (Jiang, Wu, Lu, Lu, & Xu, 1997), immunomodulatory (Jiang & Xu, 2003; Qiang, Cao, Wu, Chen, & Jiang, 2010), anti‐tumor (Sa et al., 2008), hypoglycemic effect (Fukunaga, Miura, Furuta, & Kato, 1997), etc. Phytochemical analysis showed that flavonoids, including astilbin, neoastilbin, neoisoastilbin, isoastilbin, engeletin, and isoengeletin, are the main bioactive components of *RSG* (Chen, Yin, Yi, Xu, & Chen, 2007; Zhang, Li et al., 2009; Zhang, Zhang et al., 2009). Hence, to develop modern medicament of *RSG*, the extraction and purification of total flavonoids from *RSG* attracts our interest.

Cyclodextrins (CDs) are cyclic oligosaccharides with peculiar structure of hydrophobic cavity, which can form inclusion complexes with many bioactive compounds and increase their solubility, stability, and bioavailability (Szente & Szejtli, 2004). In traditional extraction process, organic reagents, for example, ethanol–water solution, are usually the best solvent for many targeted phytochemicals extraction. In some recent studies, the encapsulation ability of β‐CD has been used to assist the extraction of bioactive ingredients from plant material (Mantegna et al., 2012; Rajha et al., 2015; Zhang et al., 2015). The method is more environmentally friendly because β‐CD aqueous solution is used instead of organic solvent. Our previous works showed that astilbin, the most dominant flavonoid in *RSG*, is a poorly soluble compound. However, β‐CD could significantly increase its solubility through complexes formation (Zhang, Cheung, & Zeng, 2013; Zhang, Nie et al., 2013).

In conventional purification processes, plant material was usually extracted by ethanol–water solvent. After removing ethanol by concentration (or further lyophilized and re‐dissolved in water), the extract was then gone through resin adsorption/desorption process for target compounds purification (Lin, Zhao, Dong, Yang, & Zhao, 2012; Ma et al., 2009). The extraction process needs large amount of organic solvent, energy, and labor. In the present study, we aimed to develop a convenient and eco‐friendly method for total flavonoids purification from *RSG*. β‐CD aqueous solution was used for extraction instead of ethanol solvent. Without ethanol, the extract could be directly used for resin adsorption, which was convenient, energy, and labor saving. The CD‐assisted extraction was optimized through defining optimal CD species, concentration, extraction temperature, and time. Resin adsorption/desorption process has proved to be an efficient technique in the field of natural products purification (Li & Chase, 2010; Sun et al., 2017). The adsorption properties of eight macroporous resins on astilbin were compared. H103 resin with the best performance was selected, and the effects of β‐CD were further studied. Finally, the method of β‐CD‐assisted extraction and resin adsorption/desorption was used to purify the total flavonoids from *RSG* with satisfied results.

## 2 MATERIALS AND METHODS

### 2.1 Chemicals and materials

Astilbin (>98%) was purified from *RSG* in our laboratory and was identified by UV, IR, MS, and NMR (Zhang, Cheung et al., 2013; Zhang, Nie et al., 2013). *RSG* sample was purchased from Kangmei Pharmaceutical Co., Ltd. (Puning city, Guangdong province, China). The material was smashed by high‐speed pulverizer and filtered through 60 mesh sieve. Eight kinds of macroporous resins (DM2, DM21, DM28, D101, DM130, X‐5, H103, 860021) were purchased from Amicogen Biopharm Co., Ltd. (Jining city, Shandong province, China). The physical properties of these resins were listed in supplemental material (Supporting information Table S1). Before using, the resins were pretreated by soaking in 95% ethanol for 24 hr first. Then, the resins were soaked in 5% HCl (v/v) and 5% NaOH (w/v) for 4 hr, respectively. Finally, the resins were washed to neutral pH with distilled water. α‐, β‐, and γ‐CD (>99%) were purchased from Jiangsu Fengyuan Biotechnology Co. Ltd. (Suqian city, Jiangsu province, China). HPLC grade acetonitrile was purchased from Anhui Tedia High Purity Solvents Co., Ltd (Anqin city, Anhui province, China). Milli‐Q water was used throughout the study. All other reagents used were analytical grade.

### 2.2 HPLC analysis

An Agilent 1,260 HPLC system (Agilent Technologies, Palo Alto, CA, USA) and a Symmetry C18 column (250 mm × 4.6 mm i.d., 5 μm) (Waters Corporation, Milford, MA, USA) were used for HPLC analysis. The mobile phase consisted of acetonitrile (A) and 0.1% acetic acid aqueous solution (B). The flow rate was 1 ml/min with linear gradient program of 0‐15 min, 16%‐20% A; 15‐40 min, 20%‐40% A. Detected wavelength was 291 nm with an injection volume of 10 μl.

### 2.3 Extraction efficiency comparison between CDs aqueous and organic solution

One‐half gram of *RSG* sample was immersed with 25 ml of α‐, β‐, and γ‐CD aqueous solution with concentration of 0.5%, 1%, and 1.5% (W/V), respectively. For comparison, one‐half gram of *RSG* sample was immersed with 25 ml of ethanol–water solution with different ethanol content (0, 10, 20, 30, 40, 50, 60, 75, and 95%, V/V). The mixture was shaken mechanically at 25°C for 30 min. The content of total flavonoids in the extract was measured by HPLC.

### 2.4 Kinetic adsorption and adsorption isotherms

Astilbin solution with concentration of 2 mg/ml was freshly prepared by dissolving 1 g of astilbin with 50 ml of 40% ethanol and then diluted to 500 ml with water in a volumetric flask. In a 100‐ml stoppered conical flask, a 50‐ml aliquot of astilbin solution was mixed with 1 g resins (wet weight after suction filtration). The flasks were shaken mechanically at 25°C. The concentration of astilbin in solution was determined spectrophotometrically at 291 nm with intervals of 30 min. The calibration curve for astilbin quantification was *Y* = 0.036*X*‐0.02,where *Y* was the absorbance and *X* was the concentration of astilbin (ranged from 1 to 30 μg/ml), *R* ^2 ^= 0.997.

The adsorption isotherms were studied at 25°C. The astilbin solution was properly diluted to concentration of 0.2, 0.4, 0.8, 1.0, 1.2, 1.6, 1.8, and 2.0 mg/ml (*C*_0_), respectively. In a 100‐ml stoppered conical flask, a 50 ml aliquot of astilbin solution was mixed with 1 g resins. The flasks were shaken mechanically at speed of 160 rpm for 3 hr to reach the equilibrium. The remaining astilbin (*C*_e_) in the solution was determined spectrophotometrically at 291 nm. The adsorption capacity of resin (*Q*_e_) was calculated with equation (1):

(1) $$Q_{e}=\frac{C_{0}-C_{e}}{m}\times V$$

where *C*_0_ and *C*_e_ are the initial and equilibrium concentration of astilbin (mg/ml), *m* and *V* are the weight of resin (g) and volume of astilbin solution (ml) used in the study, respectively. Langmuir and Freundlich models were used to simulate the adsorption isotherm data. The models are expressed as follows (Guo, Zhang, Chen, Shangguang, & Guo, 2015; Sandhu & Gu, 2013):

Langmuir model:

(2) $$C_{e}/Q_{e}=C_{e}/Q_{m}+1/\left(Q_{m}\times K_{L}\right)$$

Freundlich model:

(3) $$\text{ln}\;Q_{e}=\text{ln}\;K_{F}\;+\;\left(1/n\right)\;\times \;\text{ln}\;C_{e}$$

where *C*_e_ is the equilibrium concentration; *Q*_e_ is the adsorption capacity of resin; *Q*_m_, *K*_L_, *K*_F_, and 1/*n* are the constants of models.

### 2.5 Desorption test

Briefly, 1 g H103 resin was mixed with 50 ml of astilbin solution (2.0 mg/ml). After shaking for 3 hr to reach the adsorption equilibrium, the resin was obtained by suction filtration. Then, the resin was desorbed by 50 ml of ethanol–water solution by shaking at 160 rpm at 25 °C for 1 hr. The desorption ratio was calculated as follows:

(4) $$D\left(\%\right)\;=\;\left(C_{d}\;\times \;V\right)/\left(Q_{e}\;\times \;m\right)\;\times \;100\%$$

where *C*_d_ (mg/ml) is the concentrations of astilbin in the desorption solutions; *Q*_e_ (mg/g) is the resin adsorption capacity; *m* and *V* are the weight of resin (g) and volume of astilbin solution (ml) used in the study, respectively.

### 2.6 Effects of β‐CD on the adsorption and desorption of H103 resin

Except astilbin was dissolved in β‐CD aqueous solution, the kinetic adsorption and adsorption isotherms of H103 resin were studied the same as described in Section 2.4.

H103 resin fixed bed in a glass column was used for dynamic adsorption and desorption tests. The bed volume (BV) was 10 ml with packed length of 33 cm. The adsorption process was performed by pumping astilbin solution (1 mg/ml) through the column with flow rate 10 ml/min (1BV/min) or 5 ml/min (0.5 BV/min). Astilbin was dissolved in water, 0.5% and 1% β‐CD aqueous solution with concentration of 1 mg/ml, respectively. The astilbin content in effluent solutions was analyzed at 5 min intervals.

After dynamic adsorption, the resin was desorbed by 75% ethanol with flow rate of 5 ml/min. The concentration of astilbin in the effluent solution was analyzed at 2 min intervals.

### 2.7 Purification of total flavonoids from *RSG*

*Rhizoma Smilacis Glabrae* sample (50 g) was extracted by 750 ml of 0.5% β‐CD aqueous solution for twice. After centrifugation under 1,500 g for 5 min, the supernatant was combined and used for resin adsorption directly. The BV of H103 resin fixed bed was 160 ml with height of 51 cm. The extract was pumped by a peristaltic pump with constant flow rate of 10 ml/min. After adsorption, the fixed bed was desorbed by 4 BV of 75% ethanol with flow rate of 10 ml/min. The eluent was concentrated and then lyophilized to obtain the resin purification product.

To obtain the crude extract of *RSG* for comparison, 50 g of *RSG* sample was extracted by 750 ml of 60% ethanol for twice. The supernatant was directly lyophilized after concentration.

### 2.8 Statistical analysis

Data were expressed as the mean ± standard deviation (SD) of triplicates. Data analysis and plotting were performed with software of Origin 8.0 (Origin Lab Co., Northampton, MA, USA). One‐way ANOVA was used for statistical analysis. Differences were considered significant when *p* < 0.05.

## 3 RESULTS AND DISCUSSION

### 3.1 HPLC determination of total flavonoid content in *RSG*

**Figure 1:**
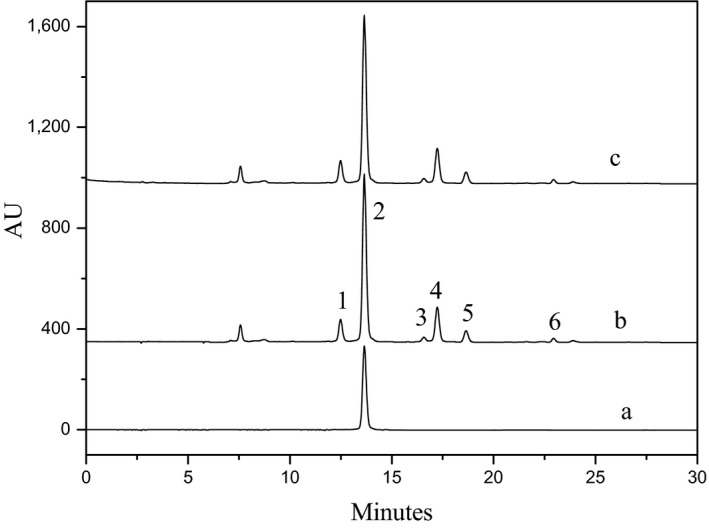
The HPLC chromatography of astilbin standard (a), *Rhizoma Smilacis Glabrae (RSG)* sample (b), and H103 resin purified product (c). Peaks: 1, neoastilbin; 2, astilbin; 3, neoisoastilbin; 4, isoastilbin; 5, engeletin; and 6, isoengeletin

The total flavonoids content of *RSG* was determined by HPLC method. Under the optimal separation conditions, we reported previously (Zhang, Cheung et al., 2013; Zhang, Nie et al., 2013), the HPLC chromatogram of *RSG* was shown in Figure 1. As can be seen, astilbin (peak 2) was the most dominant flavonoid in *RSG*. The calibration curves of astilbin were *Y* = 20.734*X*‐0.785, with correlation coefficient of 0.999, where *Y* was the peak area and *X* was concentration of astilbin (5‐500 μg/ml). Peak 1, 3, and 4 were the isomers of astilbin, namely neoastilbin, neoisoastilbin, and isoastilbin, respectively. Peak 5 and 6 were engeletin and isoengeletin (Zhang, Cheung et al., 2013; Zhang, Nie et al., 2013). All these substances are dihydroflavonols. Because many of these standard markers are market unavailable, the total flavonoid content of *RSG* was determined by substituting the area sum of peak 1‐6 into the calibration curves of astilbin. The total flavonoid content of present *RSG* sample was determined as 48.2 mg/g with astilbin content of 33.9 mg/g.

### 3.2 Extraction efficiency comparison between CDs aqueous and ethanol solution

**Figure 2:**
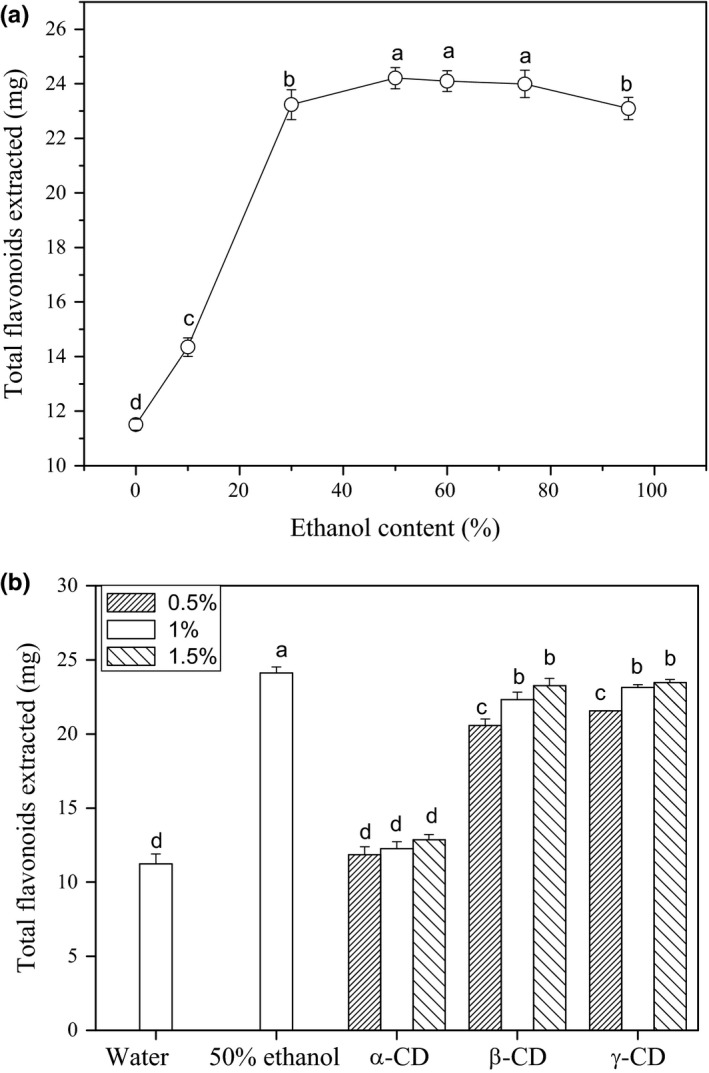
The total flavonoids extraction efficient comparison between different ethanol solvents (a) and CDs aqueous solution (b). Different letter in the graph means significant difference (ANOVA,* p* < 0.05)

Ethanol–water is usually the best solvent for flavonoids extraction from plant materials. As shown in Figure 2a, comparing with water only, the adding of ethanol in extractant notably increased the yield of total flavonoids from *RSG*. The yield quickly increased with rise of ethanol content first and then became stable. The optimal extraction solvent was 50% ethanol.

The extraction efficiency comparison between CD aqueous solution and 50% ethanol was showed in Figure 2b. As shown, when β‐ and γ‐CD were added to water, the extraction yield of total flavonoids was significantly increased. The effect was attributed to the formation of complexes between flavonoids and CDs, which significantly increases their solubility in water. As we have calculated previously, the formation constants of astilbin with β‐ and γ‐CD were 2305.9 and 995.2/M, and its solubility was increased 7.58 and 6.71 times with adding 5 mM of β‐CD and γ‐CD, respectively (Zhang, Cheung et al., 2013; Zhang, Nie et al., 2013). However, α‐CD almost had no effect on total flavonoids extraction. These differences were due to the variety of cavity size between the three CDs. The cavity diameters of α‐, β‐, and γ‐CD are 0.49, 0.62, and 0.79 nm, respectively. The finding is in accordance with our previous report that the cavity of α‐CD is too small for astilbin to enter (Zhang, Cheung et al., 2013; Zhang, Nie et al., 2013).

Although the extraction yield of total flavonoids slightly increased with the rise of β‐ and γ‐CD concentration, their extraction efficiency was slightly lower than 50% ethanol. With 1% β‐ and γ‐CD, the total flavonoids extracted from 0.5‐g *RSG* sample were 23.25 and 23.47 mg, respectively. The value was 24.1 mg for 50% ethanol. The extraction efficiency of β‐ and γ‐CD was similar. However, the market price of β‐CD is much cheaper than γ‐CD. Thus, 1% β‐CD was used to assist the extraction process in subsequent study.

### 3.3 Optimization of β‐CD‐assisted extraction

The β‐CD‐assisted extraction was optimized through defining extraction temperature, time, and liquid‐to‐solid ratio with the criterion of higher extraction yield of total flavonoids. These data were shown in Supporting information Figure S1 in supplemental material. As shown in Supporting information Figure S1A, temperature had significant effect on the extraction yield of total flavonoids. The yield increased gradually with the rise of temperature. This is because high temperatures can speed up the diffusion of flavonoids from *RSG* into aqueous solution. However, the complexation of β‐CD with astilbin is exothermic, and the rise of temperature is not good for the reaction (Zhang, Cheung et al., 2013; Zhang, Nie et al., 2013). Thus, 35°C was selected as the extraction temperature. In Supporting information Figure S1B, the total flavonoids yield increased gradually with extraction time from 10 to 40 min and then became stable. Thus, 40 min was selected as the extraction time. Supporting information Figure S1C showed the effects of liquid‐to‐solid ratio. The extraction yield of total flavonoids quickly increased with the rise of liquid‐to‐solid ratio first and then became stable. It is easy to understand that to a certain extent, the more extractant, the better mass transfer.

Based on the optimization study, the selected extraction temperature, time, and liquid‐to‐solid ratio in subsequent study were 35°C, 40 min, and 30:1, respectively.

### 3.4 Static adsorption comparison between the eight resins

**Figure 3:**
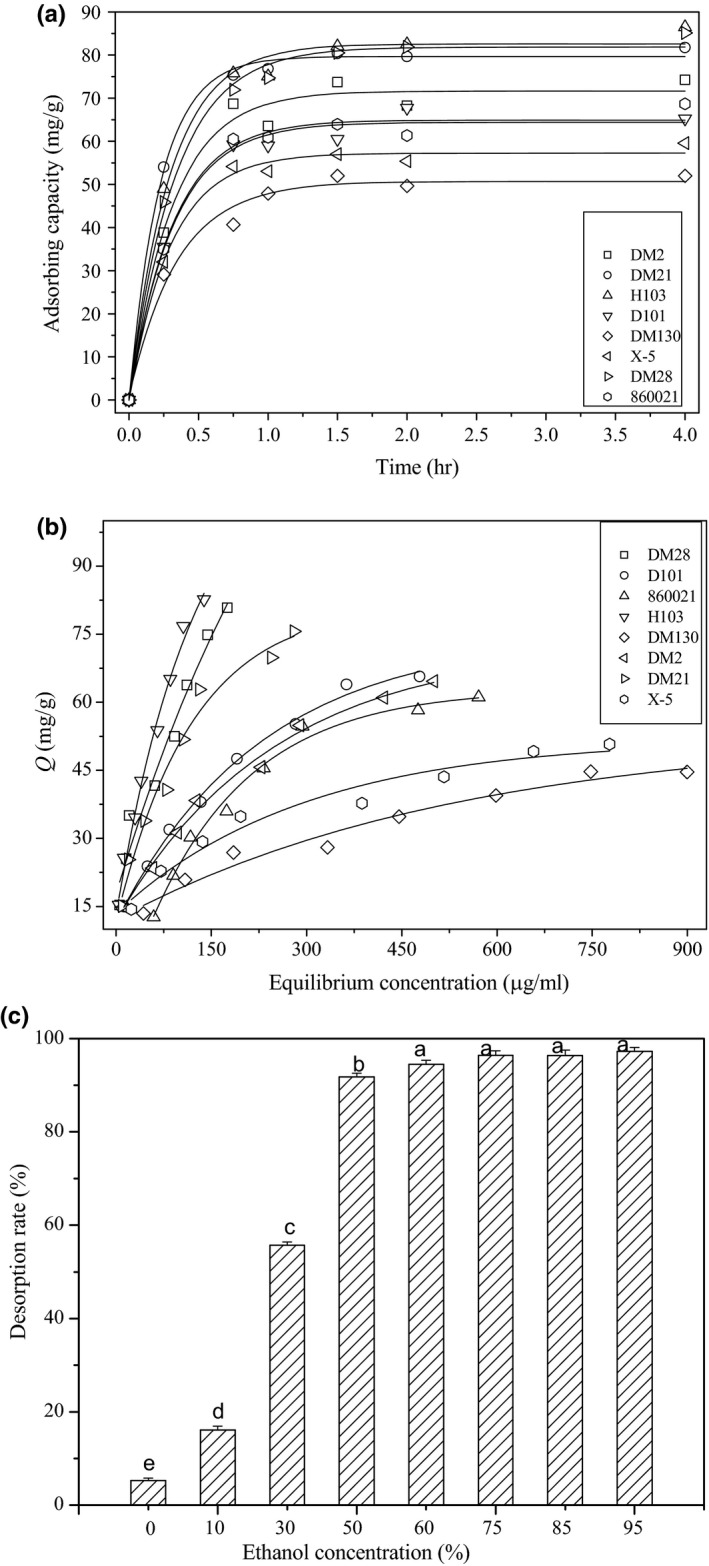
The kinetic adsorption results (a) and equilibrium adsorption isotherms (b) of the eight resins at 25°C; The desorption efficiency of different ethanol concentration (c). Different letter in the graph means significant difference (ANOVA,* p* < 0.05)

The adsorption kinetics of eight macroporous resins on astilbin was compared in Figure 3a. As shown, the adsorption rate of H103 resin was fastest among the eight resins. The adsorption capacity of all resins increased rapidly at first and then gradually reached the equilibrium at about 2 hr. Figure 3b showed the adsorption capacity of the eight resins with different equilibrium concentration of astilbin after 3 hr of adsorption at 25°C. From the adsorption isotherms, the adsorption capacity of all resins increased with the rise of astilbin equilibrium concentration. However, the adsorption properties of resins were different, which may due to their difference of surface area, pore diameter, and surface polarity. H103, DM21, and DM28 showed the best adsorption kinetics and capacity. From the physical properties listed in Supporting information Table S1, the three resins possess the biggest surface area and smallest pore diameter among the eight resins.

Langmuir and Freundlich equations are the two theoretical models frequently used for analysis of adsorption from solution. The Langmuir model describes a monolayer adsorption based on homogeneous and finite‐sorption‐site assumptions, while Freundlich model is suited for both monolayer and multilayer adsorption on heterogeneous surfaces (Guo et al., 2015). Table 1 listed the simulated parameters through the experimental data for both models. As reflected from the correlation coefficients, Freundlich model was a better model for most of the resins. In Langmuir model, parameters *Q*_m_ and *K*_L_ are constants related to the maximum adsorption capacity and affinity of adsorption, respectively. H103 resin has the biggest values of *Q*_m_ and *K*_L_, which meant that its adsorption capacity and affinity with astilbin were strongest among the eight resins. Similar results were obtained with Freundlich model. H103 resin also has the biggest values of ln*K*_F_ and 1/*n*. In the model, *K*_F_ is an indicator of adsorption capacity, and 1/*n* is an empirical constant related to the magnitude of the adsorption driving force. Thus, H103 was selected as the best resin for purification of total flavonoids from *RSG*.

**Table 1:** Fitting parameters of Langmuir and Freundlich models for the eight resins

	Langmuir model			Freundlich model		
	*Q*_m_	*K*_L_	*R* ^2^	lnKF	1/*n*	*R* ^2^
DM28	94.88	20.75	0.903	5.15	0.46	0.965
D101	79.43	9.08	0.965	4.54	0.43	0.990
860021	97.66	3.29	0.914	4.66	0.66	0.902
H103	105.04	21.36	0.915	5.38	0.50	0.990
DM130	52.94	5.30	0.964	3.86	0.39	0.977
DM2	76.86	8.62	0.962	4.45	0.41	0.981
DM21	89.77	15.35	0.967	4.95	0.46	0.975
X‐5	55.87	8.75	0.979	4.03	0.35	0.982
H103^a^	85.39	32.02	0.990	4.92	0.39	0.968
H103^b^	90.57	27.61	0.990	5.05	0.43	0.960

^a^In the presence of 1% β‐CD; ^b^In the presence of 0.5% β‐CD.

Besides adsorption, desorption is also very important to obtain the purified product from resin. Ethanol–water is the commonly used desorption solvent without toxicity. Figure 3c compared the desorption property of ethanol–water solution with different percentage of ethanol. As shown, using water only could not desorb astilbin from H103 resin. With the rise of ethanol concentration, the desorption ratio of astilbin increased quickly first and then became stable. The recovery of astilbin reached up to 96.3% when 75% ethanol was used.

### 3.5 Effects of β‐CD on the adsorption and desorption of H103 resin

**Figure 4:**
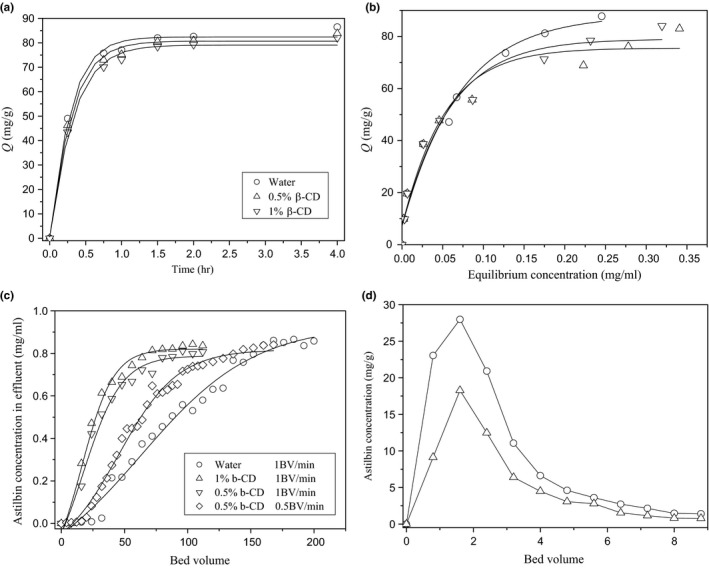
The kinetic adsorption results (a), equilibrium adsorption isotherms (b), and dynamic adsorption curve (c) of H103 resin in the presence of different concentration of β‐CD. (d) The elution curve of H103 fixed bed adsorbed astilbin in water (○) and 1% β‐CD (∆)

As β‐CD was used for extracting the total flavonoids from *RSG*, its effects on the adsorption capacity of H103 resin were further investigated. Figure 4a and b showed the kinetic adsorption results and adsorption isotherms of H103 in the presence of different concentration of β‐CD, respectively. As shown, the presence of the β‐CD would slightly decrease the adsorption rate and capacity of H103 with concentration dependent. The higher concentration of β‐CD, the more significant effects were found. Adsorption isotherms showed that the capacity of H103 decreased more with higher equilibrium concentration of astilbin. As analyzed by Freundlich model, the values of ln*K*_F_ and 1/*n* decreased with rise of β‐CD concentration (Table 1), which meant that its adsorption capacity and affinity with astilbin were decreased.

The difference was more obvious when dynamic adsorption was performed. Figure 4c showed the dynamic adsorption results of H103 in the presence and absence of β‐CD with different flow rate. As shown, the concentration of astilbin in the effluent gradually increased, which meant more and more astilbin was unadsorbed. When the concentration of astilbin in the effluent reaches 5% and 90% (or becomes stable) of its initial concentration, the point is regarded as leak point and breakthrough point, respectively. Under the flow rate of 1 BV/min, the leak point of H103 resin in water, 0.5% and 1% β‐CD appeared with BV of 32, 8, and 8, respectively. Meanwhile, the breakthrough point appeared with BV of 168, 104, and 88, respectively. These data were in accordance with static adsorption results that the presence of the β‐CD would weak the adsorption capacity of H103 to astilbin. However, when the flow rate was decreased to 0.5 BV/min, the adsorption performance of H103 was improved with leak point and breakthrough point at BV of 20 and 120, respectively.

The phenomenon may be attributed to the complexes formation of astilbin with β‐CD. Before adsorbed by H103 resin, astilbin should release from the cavity of β‐CD first. One more step was needed compared with astilbin dissolved in water only. Thus, when the flow rate was too fast, the exposure duration between astilbin and resin was too short in the column, and there is no sufficient time to complete the adsorption. The dynamic adsorption performance of H103 was significantly improved when decreased the flow rate from 1BV/min to 0.5BV/min.

After dynamic adsorption, the adsorbed astilbin on resin was desorbed by 75% ethanol. The elution curve was drawn in Figure 4d. Because the resin fixed bed adsorbed less astilbin when β‐CD was presented as showed in Figure 4c, the concentration of astilbin in eluant was different between the two experiments. Besides, there was no other difference between the two elution curves. The concentrated peak appeared at 2 BV of desorption solvent and then decreased drastically. At the site near 7 BV, the concentration of astilbin was lower than 2 mg/ml. Integration results revealed that with 4 BV of 75% ethanol, the recovery of astilbin was more than 95%.

### 3.6 Extraction and purification of total flavonoids from *RSG*

Through the conditions optimization as described before, 50 g of *RSG* sample was extracted with 750 ml of 0.5% β‐CD aqueous solution twice. The extract was adsorbed by H103 resin fixed bed and then desorbed by 75% ethanol. Table 2 showed the HPLC analysis results of the lyophilized product. As shown, the yield of resin purified product was 8.78%, with astilbin and total flavonoids content of 363.8 and 505.7 mg/g, respectively. Compared with the extract obtained directly through 50% ethanol extraction, the total flavonoids purity was 1.74 times increased with the recovery of 94.38%. The HPLC chromatogram shown in Figure 1 indicated that the chemical profile between the two products had no difference as reflected through the peak number and area. These results showed that the method of β‐CD‐assisted extraction and H103 resin adsorption/desorption was successfully used for total flavonoids purification from *RSG* sample.

**Table 2:** Yield and total flavonoids content of crude extract and H103 resin purified product of *Rhizoma Smilacis Glabrae (RSG)*

	Crude extracts	Resin purified products	Recovery (%)	Purity increase (times)
Yield (%)	16.21 ± 0. 30	8.78 ± 0.43	‐	‐
Astilbin content (mg/g)	208.9 ± 10.1	363.8 ± 9.1	94.33	1.74
Total flavonoids content^a^ (mg/g)	290.2 ± 10.7	505.7 ± 8.9	94.38	1.74

a Calculated as astilbin equivalents.

## 4 CONCLUSION

An eco‐friendly method with β‐CD‐assisted extraction and resin adsorption/desorption was developed for total flavonoids purification from *RSG* sample with satisfied results. The yield of resin purified product was 8.78%, with astilbin and total flavonoids content of 363.8 and 505.7 mg/g, respectively. Compared with the extract obtained directly through 50% ethanol extraction, the purity was 1.74 times increased with the recovery of 94.38%. In this method, *RSG* sample was extracted by 0.5% β‐CD aqueous solution and then directly used for H103 resin adsorption, which is convenient, energy, and labor saving. Except for desorption process, the method needs no organic solvent and is environmentally friendly.

## CONFLICT OF INTEREST

The authors declare that there are no conflict of interests.
