# Supplementary material for: Purification of total flavonoids from Rhizoma Smilacis Glabrae through cyclodextrin‐assisted extraction and resin adsorption
Source: Food Sci Nutr. 2019 Jan 29;7(2):449–56. doi: 10.1002/fsn3.809 (PMC6392876; doi:10.1002/fsn3.809)
Supplement: Supplementary file 1 [file FSN3-7-449-s001.docx]

**Supplemental material**

**for**

**Purification of total** **flavonoids from** ***Rhizoma Smilacis Glabrae* through cyclodextrin-assisted** **extraction and resin adsorption**

Table S1 Physical properties of the eight macroporous resins

|  | Matrix | Polarity | Surface area (m2/g) | Particle diameter (mm) | Average pore diameter (nm) |
| --- | --- | --- | --- | --- | --- |
| DM2 | SDVB | Non-polar | ≥800 | 0.31-1.21 | 5-6 |
| DM21 | SDVB | Non-polar | ≥1000 | 0.25-0.84 | 5-6 |
| DM28 | SDVB | Non-polar | ≥1200 | 0.2-0.6 | 8.5-9.5 |
| D101 | SDVB | Non-polar | 480–520 | 0.3-1.25 | 25–28 |
| DM130 | SDVB | weak-polar | 500-550 | 0.3-1.25 | 9-10 |
| X-5 | SDVB | Non-polar | 500–600 | 0.3-1.25 | 29–30 |
| H103 | SDVB | Non-polar | ≥1100 | 0.25-0.84 | 6-7 |
| 860021 | SDVB | weak-polar | 450–550 | 0.25-0.84 | 9-10 |

**Figure S1**

Figure s1. Optimization of β-CD assisted extraction, effect of temperature (A), extracting time (B) andliquid-to-solid ratio (C). Different letter in graph means significant difference (ANOVA, p < 0.05).
